# Dying in times of COVID-19: Experiences in different care settings – An online questionnaire study among bereaved relatives (the CO-LIVE study)

**DOI:** 10.1177/02692163221079698

**Published:** 2022-03-09

**Authors:** Berivan Yildiz, Ida J Korfage, Erica FE Witkamp, Anne Goossensen, Liza GG van Lent, H Roeline Pasman, Bregje D Onwuteaka-Philipsen, Masha Zee, Agnes van der Heide

**Affiliations:** 1 Erasmus MC, University Medical Center Rotterdam, Rotterdam, The Netherlands; 2 Research Centre Innovations in Care, University of Applied Sciences, Rotterdam, The Netherlands; 3 University of Humanistic Studies, Utrecht, The Netherlands; 4 Department of Medical Oncology, Erasmus MC Cancer Institute, University Medical Center, Rotterdam, The Netherlands; 5 Department of public and occupational health, Expertise Center for Palliative Care, Amsterdam UMC, VU University, Amsterdam, the Netherlands

**Keywords:** COVID-19, end of life, quality of care, death, family

## Abstract

**Background:**

The COVID-19 pandemic and restricting measures have affected end-of-life care across different settings.

**Aim:**

To compare experiences of bereaved relatives with end-of-life care for a family member or friend who died at home, in a hospital, nursing home or hospice during the pandemic.

**Design:**

An open observational online survey was developed and disseminated via social media and public fora (March–July 2020). Data were analyzed using descriptive statistics and logistic regression analyses.

**Participants:**

Individuals who lost a family member or friend in the Netherlands during the COVID-19 pandemic.

**Results:**

The questionnaire was filled out by 393 bereaved relatives who lost a family member or friend at home (*n *=* *68), in a hospital (*n *=* *114), nursing home (*n *=* *176) or hospice (*n *=* *35). Bereaved relatives of patients who died in a hospital most often evaluated medical care (79%) as sufficient, whereas medical care (54.5%) was least often evaluated as sufficient in nursing homes. Emotional support for relatives was most often evaluated as sufficient at home (67.7%) and least often in nursing homes (40.3%). Sufficient emotional support for relatives was associated with a higher likelihood to rate the place of death as appropriate. Bereaved relatives of patients who died at a place other than home and whose care was restricted due to COVID-19 were less likely to evaluate the place of death as appropriate.

**Conclusion:**

End-of-life care during the COVID-19 pandemic was evaluated least favourably in nursing homes. The quality of emotional support for relatives and whether care was restricted or not were important for assessing the place of death as appropriate.

**What is already known about the topic?**

- During the COVID-19 pandemic, restricting measures have significantly influenced end-of-life care.

**What this paper adds?**

- Bereaved relatives most often evaluated medical care as sufficient in hospitals, whereas in nursing homes medical care was least often evaluated as sufficient.
- Emotional support for relatives was most often evaluated as sufficient at home and least often in nursing homes, and was associated with a higher likelihood to rate the place of death as appropriate.
- Dying at a place other than home and care or treatment being restricted due to COVID-19 were associated with a lower likelihood to evaluate the place of death as appropriate.

**Implications for practice, theory or policy**

- We recommend to enable patients to die at home when possible, also during future pandemics.
- Our findings highlight the importance of addressing emotional care needs of relatives of patients at the end of life.

## Introduction

The COVID-19 pandemic has affected almost every aspect of healthcare provision. In particular, the provision of end-of-life care has faced unprecedented challenges due to restricting measures to prevent the spread of the virus. As a result, core values in end-of-life care, such as focusing on the individual needs and preferences of dying persons and their families have been at risk.^1^ In the Netherlands, access to institutional or specialized end-of-life care was limited.^2^ Further, the frequency and duration of allowed visits was severely restricted. These factors have impacted end-of-life care experiences of patients and their families, and increased feelings of loneliness and distress among all those involved.^1,3,4^

Qualitative studies on experiences of relatives have described the impact of restricting measures on practical and emotional aspects at the end of life, such as saying goodbye and communication with healthcare staff.^5,6^ For instance, one study among relatives showed that end-of-life communication was negatively influenced by limited availability of staff, insufficient updates regarding the patients’ condition, and not being consulted about decision-making on care and treatment.^5^ However, the impact of the pandemic on end-of-life care may vary for different healthcare settings, depending on several factors, such as the characteristics of patients and the level of visiting restrictions. A Swedish study demonstrated that patients dying in nursing homes during the COVID-19 pandemic significantly less often had retained the ability to express their will during the last week of life compared to patients dying in the hospital.^7^ Furthermore, relatives of patients dying in nursing homes were present at the time of death in 13% of the cases compared to 24% in hospitals.^7^ Another study found that healthcare professionals in Dutch hospitals and nursing homes evaluated care less favourably than healthcare professionals at home settings and hospices.^8^

Investigating bereaved relatives’ appreciation of end-of-life care among different settings is pivotal for a better understanding of the impact of the pandemic on the quality of end-of-life care. Therefore, the aim of this study was to gain insight in how bereaved relatives evaluated end-of-life care during the first wave of the pandemic in four settings: at home, in a hospital, a nursing home and a hospice. Further, we studied whether relatives in hindsight considered the place of death appropriate and how this evaluation was associated with characteristics of patients, relatives and care as provided.

## Methods

### Design and population

An open observational online questionnaire study was conducted in the Netherlands to assess experiences of end-of-life care during the first wave of the COVID-19 pandemic. The questionnaire could be filled in by individuals (⩾18 years) who had experienced the death of a family member or friend, either with or without the COVID-19 virus, between 1 March and 31 July 2020. No additional inclusion or exclusion criteria were applied. 821 individuals visited the survey page. In total, 420 relatives filled in the questionnaire, with 397 bereaved relatives completing the entire questionnaire. Relatives who provided information on the place of death of the deceased person were selected (*n *=* *393) for the present study. In this paper, ‘relatives’ is used to represent both bereaved family members and friends.

### Recruitment

The survey was circulated widely through health care organizations (hospitals, nursing homes, hospices and general practitioners), palliative care networks and personal contacts throughout the Netherlands. Organizations used their website, e-mail distribution lists, newsletters and/or social media to advocate the survey. All announcements included a link to the survey which was made in an online data collection programme (LimeSurvey).The first page of the survey included an explanation about the aim of the study with contact details of the researchers for further information. At the second page respondents were asked if they agreed if that responses were used for the purposes of the study, before voluntarily filling out the survey. Responses were anonymous unless respondents shared their email address to indicate interest in further research.

### Data collection

The questionnaire included an abbreviated version of the international Care Of the Dying Evaluation (iCODE) questionnaire that focuses on the last 2 days of life and bereavement period, and asks about the characteristics of the care that was provided, respondents’ appreciation of the care and communication with healthcare staff, and family support.^9^ Self-developed questions about the impact of COVID-19 related measures were asked. Options such as ‘don’t know’ or ‘not applicable’ were included for respondents who were not allowed or not able to visit the dying person in the last days of life. In addition, free-text space was available for additional comments at the end of the survey. The questionnaire included nine pages, with 3–14 items on each page. In total, 51 items were included in the questionnaire. Bereaved relatives were able to review and change their answers through a ‘go back’ button.

#### Patients’ and bereaved relatives’ characteristics

Respondents were asked to provide information about patients’ and bereaved relatives’ sex, age and whether they had COVID-19. Relatives were also asked to indicate whether the deceased person had a chronic serious illness (cancer, heart disease, lung disease, diabetes, dementia or other) and whether the deceased had been suffering from several symptoms in the last 2 days of life (shortness of breath, pain and restlessness).

#### Care characteristics

Relatives were asked to indicate whether they thought the medical, nursing and personal care for the deceased person had been sufficient. We also asked their opinion on the level of emotional and spiritual care. Bereaved relatives were further asked to indicate whether they considered the place of death as the appropriate place to die for the deceased person.

#### Circumstances and restrictions

Circumstances asked for whether the relative had been involved in care and treatment decisions, had been told by a healthcare professional that death was near, and had been told what to expect at the moment of death. Relatives were also asked to indicate whether care or treatment was restricted due to COVID-19 and related measures, whether they were allowed to help with care for the deceased person after death and whether visitors were allowed in the last 2 days of life.

### Analyses

Descriptive statistics were used to summarize characteristics of end-of-life care as assessed by bereaved relatives. To test the statistical significance of differences between settings, chi-square tests were used for categorical variables and Analysis Of Variance (ANOVA) for continuous variables. Univariable and multivariable logistic regression analyses were performed to investigate the association of characteristics of patients and bereaved relatives, end-of-life care, circumstances and COVID-19 restrictions (independent variables), with considering the place of death as appropriate (dependent variable). Significant independent variables (*p *< 0.05) in the univariate analyses were included in multivariable logistic analyses. A dichotomous variable was computed for the assessment of the appropriate place of death (yes vs no/don’t know/hesitant). In order to investigate whether differences exist between settings in effect estimates from the multivariable regression analysis, interaction terms with setting and statistically significant variables (e.g. sufficient emotional support*setting) were added to the multivariable logistic regression model. The omnibus tests of model coefficients was used to indicate whether the model with interaction terms had a better fit than the model without interaction terms. Missing observations were not imputed; numbers of missing observations are reported in the tables. All analyses were performed with SPSS 25.0 statistical software.

A dichotomous variable was computed for sufficient care (sufficient vs insufficient). Care was considered sufficient when the respondent indicated to agree or strongly agree with a statement that it was sufficient. Emotional support for relatives was considered sufficient when the participant indicated the support to be moderate, good or excellent. Being involved in care and treatment decisions (very involved, moderately involved, not involved), and care restricted due to COVID-19 (yes, no, don’t know) were handled as categorical variables. Being told that death was near (yes/no) and having been told what to expect of the moment of death (yes/no) were used as dichotomous variables. Having been allowed to help with care for the deceased person after death (yes, no, don’t know) and allowance of visitors in the last 2 days of life (yes, without restrictions, yes, with restrictions, no) were handled as categorical variables.

Additional comments on the appropriate place of death were ordered according to setting, to gain deeper insight into the reasons whether a place was appropriate or not.

### Ethical considerations

Respondents explicitly had to consent to their answers being used for research, before filling in the survey. Personal information of respondents (e-mail address) was stored in a separate location with access for authorized researchers only. No other personal data was collected. The research proposal was reviewed by the Medical Ethics Committee Erasmus MC of Rotterdam, The Netherlands, which assessed that the rules laid down in the Medical Research Involving Human Subjects Act did not apply (MEC-2020-0254).

## Results

### Patients’ and bereaved relatives’ characteristics

Patients who died in the hospital were more often male (64.0%) and younger of age (mean age 72.7) than patients who died at home, in a nursing home or hospice. Those who died in the hospital more often had COVID-19 (75.4%) and shortness of breath (71.2%). Patients who died at home were more often diagnosed with cancer (45.6%), whereas patients who died in a nursing home more often had dementia (62.5%). Patients who died in a nursing home suffered most often from pain (57.2%) and restlessness (67.5%). In all settings, bereaved relatives were most often female who reported on the death of their parent (Table 1).

**Table 1.** Patients’ and bereaved relatives’ background characteristics according to place of death.^a^

	Home, *n* (%)	Hospital, *n* (%)	Nursing home, *n* (%)	Hospice, *n* (%)	*p*-value
	68 (17.3)	114 (29.0)	176 (44.8)	35 (8.9)	
Patient characteristics					
*Sex*					
Female	28 (41.2)	38 (33.3)	101 (57.4)	19 (54.3)	**0.001**
Male	35 (51.5)	73 (64.0)	70 (39.8)	16 (45.7)	
Other/unknown	5 (7.4)	3 (2.6)	5 (2.8)	0	
*Age, mean (SD, range)*	76.6 (13.1, 79.0)	72.7 (17.0, 96.0)	84.5 (9.2, 78.0)	81.5 (11.1, 52.0)	0.232
*COVID-19*					
(Probably) yes	12 (17.6)	86 (75.4)	109 (61.9)	15 (42.9)	**0.000**
(Probably) no	55 (80.9)	26 (22.8)	64 (36.4)	20 (57.1)	
Unknown	1 (1.5)	2 (1.8)	3 (1.7)	0	
*Serious (chronic) illness* ^b^					
Cancer	31 (45.6)	18 (15.8)	18 (10.2)	12 (34.3)	**0.000**
Heart disease	15 (22.1)	27 (23.7)	21 (11.9)	8 (22.9)	**0.042**
Lung disease	8 (11.8)	27 (23.7)	21 (11.9)	9 (25.7)	**0.017**
Diabetes	4 (5.9)	14 (12.3)	24 (13.6)	2 (5.7)	0.243
Dementia	5 (7.4)	7 (6.1)	110 (62.5)	10 (28.6)	**0.000**
Other	20 (29.4)	27 (23.7)	48 (27.3)	9 (25.7)	0.843
*Symptoms in the last 2 days of life*					
Shortness of breath	31 (47.0)	79 (71.2)	93 (53.4)	18 (52.9)	**0.005**
Pain	36 (56.3)	45 (40.9)	95(57.2)	19 (55.9)	**0.048**
Restlessness	36 (55.4)	73 (47.7)	114 (67.5)	17 (50.0)	0.128
Bereaved relatives characteristics					
*Sex*					
Female	52 (80.0)	96 (85.7)	136 (80.0)	25 (75.8)	0.500
*Age, mean (SD, range)*	52.8 (14.8, 66.0)	53.3 (12.2, 58.0)	57.2 (12.1, 65.0)	57.2 (12.1, 65.0)	0.216
*Relation to patient*					
Child	28 (41.8)	59 (52.2)	106 (60.2)	21 (60.0)	0.079
Partner	10 (14.9)	20 (17.7)	18 (10.2)	5 (14.3)	
Other family member	25 (37.3)	28 (24.8)	45 (25.6)	9 (25.7)	
Friend	4 (6.0)	6 (5.3)	3 (1.7)	0	
Other	0	0	4 (2.3)	0	
*COVID-19*					
(Probably) yes	3 (4.6)	12 (10.7)	2 (1.2)	1 (3.0)	**0.000**
(Probably) no	58 (89.2)	80 (71.5)	160 (93.0)	29 (87.9)	
Unknown	4 (6.2)	20 (17.9)	10 (5.8)	3 (9.1)	

a Missing observations: age 6, shortness of breath 8, pain 19, restlessness 17. Bereaved relatives characteristics: sex 13, age 37, relation to patient 2, COVID-19 11.

b More than one answer possible.

P-values marked with bold indicate statistically significant differences between settings.

### Care characteristics

Bereaved relatives of patients who died in a hospital most often evaluated medical care (78.9%) as sufficient, whereas medical care was least often evaluated as sufficient in nursing homes (54.5%). In addition, emotional support for relatives was most often evaluated as sufficient at home (67.7%), followed by the hospital (66.7%), hospice (62.9%) and nursing home (40.3%). Patients’ spiritual needs were most often evaluated as having been sufficiently addressed in hospices (45.7%), and least often in nursing homes (33.5%) and at home (33.3%) (Table 2).

**Table 2.** Description of circumstances and care provided and its evaluation by bereaved relatives.^a^

	Home (*n *=* *68)	Hospital (*n *=* *114)	Nursing home (*n *=* *176)	Hospice (*n *=* *35)	*p*-value
	*n* (%)	*n* (%)	*n* (%)	*n* (%)	
Care characteristics					
Sufficient medical care^b^	49 (73.1)	90 (78.9)	96 (54.5)	23 (65.7)	**0.000**
Sufficient nursing care	42 (63.6)	86 (75.4)	114 (64.8)	23 (65.7)	0.223
Sufficient personal care	44 (65.7)	81 (71.7)	108 (61.7)	24 (68.6)	0.422
Sufficient attention for spiritual needs	21 (33.3)	43 (39.8)	57 (33.5)	16 (45.7)	0.438
Sufficient emotional support forrelatives	44 (67.7)	74 (66.7)	71 (40.3)	22 (62.9)	**0.037**
Circumstances					
*Being involved in care and treatment decisions*					
Very involved	38 (56.7)	52 (46.0)	66 (37.5)	14 (40.0)	0.084
Moderately involved	12 (17.9)	26 (23.0)	57 (32.4)	13 (37.1)	
Not involved	17 (25.4)	35 (31.0)	53 (30.1)	8 (22.9)	
*Relatives were told that death was near*					
Yes	52 (80.0)	88 (77.2)	138 (80.2)	32 (91.4)	0.328
No	13 (20.0)	26 (22.8)	34 (19.8)	3 (8.6)	
*Relatives were explained what to expect of the moment of death*					
Yes	30 (46.9)	53 (47.3)	58 (33.9)	20 (57.1)	**0.020**
No	34 (53.1)	59 (52.7)	113 (66.1)	15 (42.9)	
*Died at the appropriate place*					
Yes	62 (93.9)	67 (59.8)	117 (68.4)	25 (71.4)	**0.000**
No	1 (1.5)	17 (15.2)	27 (15.8)	5 (14.3)	
Hesitant	3 (4.4)	17 (15.2)	17 (9.9)	2 (5.7)	
Don’t know	0	11 (9.8)	10 (5.8)	3 (8.6)	
Restrictions					
*Visitors allowed in last two days*					
Yes, without restrictions	31 (48.4)	27 (23.7)	18 (10.3)	10 (28.6)	**0.000**
Yes, with restrictions	23 (35.9)	63 (55.3)	118 (67.8)	19 (54.3)	
No	10 (15.6)	24 (21.1)	38 (21.8)	6 (17.1)	
*Relatives allowed to help with care after death*					
Yes	34 (52.3)	21 (18.6)	39 (22.9)	16 (45.7)	**0.000**
No	21 (32.3)	67 (59.3)	109 (64.1)	13 (37.1)	
Don’t know	10 (15.4)	25 (22.1)	22 (12.9)	6 (17.1)	
*Care or treatment restricted due to COVID-19 pandemic*					
No	36 (54.5)	52 (46.0)	52 (29.5)	17 (48.6)	**0.003**
Yes	26 (39.4)	51 (45.1)	114 (64.8)	17 (48.6)	
Don’t know	4 (6.1)	10 (8.8)	10 (5.7)	1 (2.9)	

a Missing observations: sufficient medical care 4, sufficient nursing care 2, sufficient personal care 2, sufficient attention for spiritual needs 17, sufficient emotional support for relatives 11, being involved in care and treatment decisions 2, relatives were told that death was near 7, relatives were told what to expect of the moment of death 11, died at the appropriate place 9, visitors allowed in last 2 days 6, relatives allowed to help with care after death 10, care of treatment restricted 3.

b Care is considered sufficient when the respondent indicated to agree or strongly agree with a statement that it was sufficient.

P-values marked with bold indicate statistically significant differences between settings.

### Circumstances and restrictions

In nursing homes, relatives were least often told what to expect of the moment of death (33.9%), whereas in hospices this was most often the case (57.1%). Visiting restrictions (i.e. no visits allowed or the number of visitors or duration of visits were limited) in the last 2 days of life of the patient were most common in nursing homes (89.6%) and hospitals (76.5%). Relatives of patients who died in nursing homes more often indicated that care or treatment was restricted due to the pandemic (64.8%), whereas for patients who died at home this was least often the case (39.4%). Relatives of patients who died in a hospital were least involved in care and treatment decisions (31.0%), while relatives of patients who died at home were most often involved (56.7%). In nursing homes, relatives were most often not allowed to help with care after death (64.1%) (Table 2).

### Appropriateness of the place of death

Bereaved relatives of patients who died at home most often thought that this place of death was appropriate (93.9%), whereas bereaved relatives of patients who died in the hospital least often thought that the deceased person died at the appropriate place (59.8%). Multivariable analyses showed that bereaved relatives of patients who died in the hospital (OR 0.11, 0.03–0.44), nursing home (OR 0.24, 0.06–0.90) and hospice (OR 0.17, 0.03–0.83) were less likely to rate the place of death as appropriate, as compared to patients who died at home. Moreover, bereaved relatives who indicated to have received sufficient emotional support were more likely to rate the place of death of the deceased person as appropriate (OR 3.33, 1.60–6.96). Restriction of care or treatment due to the pandemic was associated with a lower likelihood to rate the place of death as appropriate (0.30, 0.15–0.61). We found no evidence that effect estimates were different between settings (omnibus test of model with interaction terms; *p *=* *0.37). Table 4 shows that bereaved relatives had different reasons to assess the place of death as appropriate or not (Tables 3 and 4).

**Table 3.** Patient and relatives characteristics, care characteristics, circumstances and restrictions related to the appropriateness of the place of death during the first wave of the COVID-19 pandemic.

	Died at the appropriate place	OR (95% CI) Univariate	OR (95% CI) Multivariate
	*n* (%)		
Patient characteristics			
*Sex*			
Male (*n *=* *194)	132 (69.5)	1	
Female (*n *=* *186)	128 (70.7)	1.06 (0.68–1.66)	
Other/unknown		2.42 (0.52–11.25)	
*Age (years)*		1.01 (0.99–1.03)	
*COVID-19*			
(Probably) no (*n = *222)	132 (81.5)	1	1
(Probably) yes (*n *=* *165)	134 (61.8)	**0.37 (0.23–0.59)**	0.57 (0.28**–**1.15)
*Serious (chronic) illness*			
Cancer (*n *=* *79)	67 (85.9)	**3.05 (1.54–6.01)**	1.43 (0.57**–**3.58)
Heart disease (*n *=* *71)	44 (62.0)	0.62 (0.36**–**1.06)	
Lung disease (*n *=* *65)	38 (58.8)	**0.52 (0.30–0.90)**	0.54 (0.26**–**1.12)
Diabetes (*n *=* *44)	33 (75.0)	1.29 (0.63**–**2.64)	
Dementia (*n *=* *132)	89 (67.9)	0.83 (0.52**–**1.31)	
Other (*n *=* *104)	102 (98.1)	0.65 (0.40**–**1.05)	
*Symptoms in the last 2 days of life*			
Shortness of breath			
No (164)	127 (78.4)	1	1
Yes (*n *=* *221)	143 (65.9)	**0.53 (0.33–0.85)**	0.83 (0.45**–**1.54)
Pain			
*No* (*n = *179)	118 (67.0)	1	
Yes (*n *=* *195)	145 (75.5)	1.52 (0.96**–**2.39)	
Restlessness			
*No* (*n *=* *137)	102 (76.7)	1	
Yes (*n *=* *240)	162 (68.1)	0.65 (0.40**–**1.06)	
Bereaved relatives characteristics			
*Sex*			
Male (*n *=* *71)	49 (70.0)	1	
Female (*n *=* *309)	215 (70.5)	1.02 (0.58**–**1.81)	
*Age (years)*		1.01 (0.99**–**1.03)	
*Relation to patient*			
Child (*n *=* *214)	150 (71.4)	1	
Partner (*n *=* *53)	38 (73.1)	1.09 (0.55**–**2.15)	
Other family member (*n *=* *107)	72 (69.2)	0.90 (0.54**–**1.50)	
Friend (*n *=* *13)	9 (75.0)	1.20 (0.31**–**4.59)	
Other (*n *=* *4)	1 (25.0)	0.13 (0.01**–**1.31)	
*COVID-19*			
(Probably) no (*n *=* *327)	221 (68.6)	1	
(Probably) yes (*n *=* *18)	13 (72.2)	1.19 (0.41**–**3.43)	
Don’t know (*n *=* *37)	31 (83.8)	2.36 (0.96**–**5.84)	
Care characteristics			
*Place of death*			
Home (*n *=* *68)	62 (93.9)	1	1
Hospital (*n *=* *114)	67 (59.8)	**0.10 (0.03–0.28)**	**0.11 (0.03–0.44)**
Nursing home (*n *=* *176)	117 (68.4)	**0.14 (0.05–0.40)**	**0.24 (0.06–0.90)**
Hospice (*n *=* *35)	25 (71.4)	**0.16 (0.04–0.56)**	**0.17 (0.03–0.83)**
*Sufficient medical care* (*n *=* *258)	199 (77.1)	**2.74 (1.73–4.344)**	1.53 (0.79**–**2.95)
*Sufficient nursing care* (*n *=* *265)	205 (78.5)	**3.21 (2.02–5.10)**	1.99 (0.84**–**4.74)
*Sufficient personal care* (*n *=* *257)	198 (78.0)	**2.80 (1.77–4.42)**	0.99 (0.46**–**2.38)
*Sufficient attention for spiritual needs of patient* (*n *=* *137)	113 (83.1)	**2.77 (1.64–4.66)**	1.50 (0.78**–**2.88)
*Sufficient emotional support for relative* (*n *=* *313)	242 (78.1)	**5.61 (3.21–9.83)**	**3.33 (1.60–6.96)**
Restrictions			
*Visitors allowed in last 2 days*			
Yes, without restrictions (*n *=* *86)	74 (86.0)	1	1
Yes, with restrictions (*n *=* *223)	156 (70.9)	**0.40 (0.20–0.78)**	0.86 (0.36**–**2.06)
No (*n *=* *78)	38 (50.7)	**0.17 (0.08–0.36)**	0.71 (0.25**–**2.02)
*Relatives allowed to help with care after death*			
Yes (*n *=* *110)	95 (86.4)	1	1
No (*n *=* *210)	128 (62.1)	**0.26 (0.14–0.48)**	0.72 (0.32**–**1.56)
Don’t know (*n *=* *63)	43 (69.4)	**0.36 (0.17–0.77)**	0.56 (0.21**–**1.51)
*Care or treatment restricted due to COVID-19 pandemic*			
No (*n *=* *157)	135 (87.7)	1	1
Yes (*n *=* *208)	120 (58.3)	**0.20 (0.11–0.34)**	**0.30 (0.15–0.61)**
Don’t know (*n *=* *25)	15 (68.2)	**0.30 (0.11–0.83)**	0.58 (0.16**–**2.13)
Circumstances			
*Being involved in care and treatment decisions*			
Very involved (*n *=* *170)	136 (81.0)	**1**	1
Moderately involved (*n *=* *108)	75 (70.8)	0.57 (0.32**–**1.01)	1.47 (0.69**–**3.13)
Not involved (*n *=* *113)	59 (54.1)	**0.28 (0.16–0.48)**	1.03 (0.48**–**2.18)
*Relatives were told that death was near*			
Yes (*n *=* *310)	229 (74.4)	1	1
No (*n *=* *76)	40 (54.8)	**0.42 (0.25–0.71)**	0.75 (0.36**–**1.58)
*Relatives were explained what to expect of the moment of death*			
Yes (*n *=* *161)	128 (81.0)	1	
No (*n *=* *221)	138 (63.3)	**0.40 (0.25–0.66)**	0.86 (0.45**–**1.61)

Effect estimates marked with bold indicate statistical significance.

**Table 4.** Experiences of bereaved relatives about the appropriate place of death, stratified by setting.

**Home (*n *=* *68)**	**Yes, it was the right place (*n *=* *62)**
	This was her own wish
	She died at home, but without restricting measures she would have liked to be in a hospice. But home was fine too
	She wanted to die at home, it was however distressing that because of corona her own children and grandchildren who all live abroad could not be there. She died without her family around her.
	**Hesitant (*n *=* *3)**
	Actually home was a good place, but without corona, she would have stayed in a hospice. Then the care that we provided would have been less for us. Now, the last phase was more burdensome for us in terms of providing care compared to when there was no corona.
**Hospital (*n *=* *114)**	**Yes, it was the right place (*n *=* *67)**
	My partner died in the hospital, where he was already being treated.
	We had no other choice, it just got bad really fast, especially his breathing was deteriorating very fast. The room on hospital ward [name hospital ward] was at that moment the place where it would happen. Any other place would not have changed the situation.
	**No, it was not the right place (*n *=* *17)**
	There was no other choice. We were not allowed to visit until my partner had died. I think it should have been allowed to die peacefully in the presence of his loved ones.
	He would have preferred to die at home. He was already ill and (..), the hospitalization was necessary because of Corona but has kept us away from our father during most of the time in his last week . . . We would of course have preferred to surround him with our love and care and die in our arms instead of with a nurse. This remains very painful ..I think also for the nurse who cared for him. We were just too late . . .. We received a phone call at half past seven and fifteen minutes later it had already happened. . ..
	She wanted to die at home
	**Hesitant (*n *=* *17)**
	It probably had been better for him to not go to the hospital. It was estimated that the complication could be treated, but how realistic was that?
	She would have preferred to be at home, but she had to stay in the hospital for care.
	Don’t **know (*n *=* *11)**
	Medical facilities and care were available here [in the hospital]. The disadvantage, of course, were the restrictions in supporting our father.
	Of course, that is never the right place. But they let him go as comfortably as possible.
**Nursing home (*n *=* *176)**	**Yes, it was the right place (*n *=* *117)**
	This had been her home for the past 2.5 years.
	My mother was 87 and had dementia since several years. However, she still recognized her children and grandchildren. The place was okay (her room in the care facility) but the conditions were not.
	**No, it was not the right place (*n *=* *27)**
	I would have wanted her to die at home.
	I could not be with my husband when he was still conscious. I was allowed to be there when it was clear that he was going to die, even though it had been clear for several days that things were not going well.
	If we had known this beforehand we would have taken him home. We were not allowed to go to him when things were bad.
	**Hesitant (*n *=* *17)**
	My father did die in the right place but because we were allowed to visit him in such a limited way I wonder if this was the right place. If we had admitted him to the hospital, we would have been allowed to visit more and he would not have had to die alone. However, we did not make this choice because my father wanted to stay in his own apartment.
**Hospice (*n *=* *35)**	**Yes, it was the right place (*n *=* *25)**
	My sister indicated earlier in the process that she was ‘happy’ to die in hospice.
	**No, it was not the right place (*n *=* *5)**
	We asked several times for admission to the hospital.
	Don’t **know (*n *=* *3)**
	I didn’t want him to die alone in his room.

## Discussion

### Summary

In this study, we assessed experiences of bereaved relatives with end-of-life care for a family member or friend who died during the COVID-19 pandemic. When comparing different settings we found some differences in their evaluation of care. Bereaved relatives of patients who died in a hospital most often evaluated medical care as sufficient. Emotional care for relatives and medical care in nursing homes was least often evaluated as sufficient, whereas emotional care for relatives was most often evaluated as sufficient at home. Bereaved relatives of patients who died at a place other than home, and whose care or treatment was restricted due to COVID-19 were less likely to evaluate the place of death as appropriate. In addition, sufficient emotional support for bereaved relatives was associated with a higher likelihood to rate the place of death as appropriate.

### Strengths and limitations

This study has several strengths and limitations. One strength relates to the comprehensive online survey which enabled data collection on many important aspects of end-of-life care, including assessment of the appropriate place of death. In addition, the online survey was filled in by bereaved relatives who had recently lost a family member or friend in differing settings which enabled comparison of end-of-life care between settings.

A limitation of this study is that due to its design it does not allow to assess causal relationships. It may be that differences between settings already existed before the pandemic. Bereaved relatives have previously been shown to tend to rate palliative care provided at home most positively, followed by care provided by hospices.^10^ Another study showed that relatives rated the care for patients who died in a hospice more favourably than the care received by people who died in the hospital.^11^ However, our study aimed to provide insight in differences between settings in the evaluation of end-of-life care during the pandemic since these are scarce.^12^ Another limitation is that our results may have been prone to selection bias, for example, of relatives with particularly negative experiences.

### Differences between settings

Medical care was most often evaluated as sufficient by relatives of patients dying in hospitals, but they were relatively more often not allowed to help with care after death. However, almost all aspects of care were least often evaluated as sufficient by relatives of patients who had died in nursing homes. Emotional support for relatives was also least often rated as sufficient in nursing homes, while at home and in hospices communication aspects were most often evaluated as sufficient. These findings are probably at least partly due to visiting restrictions. Several previous qualitative studies demonstrated the negative impact of restrictions on end-of-life communication, involving for example, relatives being insufficiently informed about the patient’s deteriorating condition.^5,6^ Another study showed that being unable to visit had an impact on relatives’ preparedness for patients’ death and aggravated their distress, especially among relatives who lost a family member or friend in a nursing home or hospital.^13^

It has been reported from countries all over the world that many nursing homes had poor availability of protective equipment, healthcare staff that was insufficiently trained in how to protect themselves and residents, and intensified emotions such as fear, stress and panic among both residents and healthcare staff existed.^14,15^ This may have led to healthcare professionals being less able to support patients who died in a nursing home and their relatives. In the Netherlands, healthcare staff in nursing homes often had insufficient time to call relatives by telephone, to provide an update about the patient or to provide support to relatives.^2^

Relatives of patients dying in nursing homes more often evaluated several aspects of care as insufficient compared to relatives of patients dying in the other settings. Few studies have compared the quality of end-of-life care between different settings before the pandemic. It is, however, well known that nursing homes face many challenges in providing high quality end-of-life care.^16,17^ One study in the Netherlands found that care in nursing homes was rated less highly by relatives compared to care received at home or in a hospice.^10^ In another study among 34 Dutch nursing homes, relatives reported unpleasant experiences regarding neglect (negligence in tailored care and information) and lack of respect (being insensitive towards resident and family).^18^ Other quantitative and qualitative studies among relatives have found suboptimal control of symptoms and suboptimal communication with and emotional support for residents and families.^19–22^ Together with our findings, these results emphasize the need for improving end-of-life care in nursing homes.^17,20,23–25^

Differences in evaluation of care between settings may also be explained by characteristics of bereaved relatives. Studies have demonstrated that sociodemographic characteristics such as age and gender may influence healthcare satisfaction.^26,27^ In our study, bereaved relatives of patients dying in nursing homes were relatively younger and more often women than bereaved relatives who evaluated care in the other settings. Although findings in the literature are inconsistent, some studies have demonstrated that younger people and women tend to be less satisfied with healthcare compared to older people and men, respectively.^28^ In particular, one study showed that male relatives were more likely to report being adequately supported during the last days of life during the COVID-19 pandemic.^13^

### Appropriateness of the place of death

Sufficient emotional support for relatives was associated with a higher likelihood to rate the place of death as appropriate. This may indicate that relatives consider the relational and possibly existential meaning of care for a dying person more important than medical or nursing care when assessing the appropriate place of death. It is known that patients in hospitals and their families particularly value feeling listened to and being treated with compassion by healthcare professionals.^29^ It is plausible that this important aspect has less often been prioritized in times of COVID-19, as recent studies in the United Kingdom also found that 30% of bereaved relatives indicated emotional support provided by healthcare professionals to be insufficient, and relatives highlighted their need for emotional support when their the deceased person was at the end of life.^6,13^

Bereaved relatives of patients who died at a place other than home were less likely to rate the place of death as appropriate. Especially, bereaved relatives of patients who died in the hospital and hospice were least likely to rate the place of death as appropriate. An explanation may be that the pandemic pushed bereaved relatives’ preferences for appropriate place of death outside healthcare settings, due to concerns about restricted visiting, fear of infection and motivation to reduce pressure on hospital services.^12^ However, it is well known that many people prefer to die at home.^30–32^ Place of death has therefore become a key indicator of the quality of end-of-life care.^33^ Nevertheless, dying in nursing homes, hospices, hospitals and other in-patient institutions is still common and sometimes unavoidable, due to a variety of personal and medical reasons.^10,34^

### Implications for practice

This study showed that bereaved relatives of patients who died at a place other than home were less likely to rate the place of death as appropriate. Since most aspects of end-of-life care at home were evaluated as sufficient by bereaved relatives, specific attention should be given to enable patients to die at home, also during future pandemics. Dying at home, but also dying in other settings requires sufficiently trained healthcare staff.^12,25^ Access to specialist palliative care should be widely available, also to address the emotional care needs of relatives.^35^ Psychosocial caregivers should not too easily be denied access to healthcare settings, which may also hold for volunteers who can also offer practical, emotional and social support to patients and their relatives.^36^

## Conclusion

In conclusion, we found differences in bereaved relatives’ evaluation of care between settings during the pandemic. End-of-life care during the COVID-19 pandemic was evaluated least favourably in nursing homes. Emotional support for relatives, care restrictions and place of death were important factors influencing their assessment of the appropriate place of death. During future pandemics attention should be given to high quality end-of-life care which addresses emotional needs of relatives as well.
